# Evaluation of an Experimental Web-based Educational Module on Opioid-related Occupational Safety Among Police Officers: Protocol for a Randomized Pragmatic Trial to Minimize Barriers to Overdose Response

**DOI:** 10.2196/33451

**Published:** 2022-02-25

**Authors:** Janie Simmons, Luther Elliott, Alex S Bennett, Leo Beletsky, Sonali Rajan, Brad Anders, Nicole Dastparvardeh

**Affiliations:** 1 Department of Social and Behavioral Sciences School of Global Public Health New York University New York, NY United States; 2 School of Law and Bouvé College of Health Sciences Northeastern University Boston, MA United States; 3 Department of Health and Behavior Studies Teachers College Columbia University New York, NY United States; 4 NDRI-USA New York, NY United States

**Keywords:** occupational health, law enforcement, police/education, naloxone, opioid overdose prevention and response training, online education, opioids, occupational risk

## Abstract

**Background:**

As drug-related morbidity and mortality continue to surge, police officers are on the front lines of the North American overdose (OD) crisis. Drug law enforcement shapes health risks among people who use drugs (PWUD), while also impacting the occupational health and wellness of officers. Effective interventions to align law enforcement practices with public health and occupational safety goals remain underresearched.

**Objective:**

The Opioids and Police Safety Study (OPS) aims to shift police practices relating to PWUD. It adapts and evaluates the relative effectiveness of a curriculum that bundles content on public health promotion with occupational risk reduction (ORR) to supplement a web-based OD response and naloxone training platform (GetNaloxoneNow.org, or GNN). This novel approach has the potential to improve public health and occupational safety practices, including using naloxone to reverse ODs, referring PWUD to treatment and other supportive services, and avoiding syringe confiscation.

**Methods:**

This longitudinal study uses a randomized pragmatic trial design. A sample of 300 active-duty police officers from select counties in Pennsylvania, Vermont, and New Hampshire with high OD fatality rates will be randomized (n=150 each) to either the experimental arm (GNN + OPS) or the control arm (GNN + COVID-19 ORR). A pre- and posttraining survey will be administered to all 300 officers, after which they will be administered quarterly surveys for 12 months. A subsample of police officers will also be qualitatively followed in a simultaneous embedded mixed-methods approach. Research ethics approval was obtained from the New York University Institutional Review Board.

**Results:**

Results will provide an understanding of the experiences, knowledge, and perceptions of this sample of law enforcement personnel. Generalized linear models will be used to analyze differences in key behavioral outcomes between the participants in each of the 2 study arms and across multiple time points (anticipated minimum effect size to be detected, d=0.50). Findings will be disseminated widely, and the training products will be available nationally once the study is completed.

**Conclusions:**

The OPS is the first study to longitudinally assess the impact of a web-based opioid-related ORR intervention for law enforcement in the U.S. Our randomized pragmatic clinical trial aims to remove barriers to life-saving police engagement with PWUD/people who inject drugs by focusing both on the safety of law enforcement and evidence-based and best practices for working with persons at risk of an opioid OD. Our simultaneous embedded mixed-methods approach will provide empirical evaluation of the diffusion of the naloxone-based response among law enforcement.

**Trial Registration:**

ClinicalTrail.gov NCT05008523; https://clinicaltrials.gov/show/NCT05008523

**International Registered Report Identifier (IRRID):**

DERR1-10.2196/33451

## Introduction

### Background

Accidental opioid-involved overdose (OD) mortalities in the U.S. have escalated at an alarming pace, with 81,000 OD deaths recorded in 2019-2020, the highest number ever recorded in a 12-month period. Deaths due to synthetic opioids, primarily illicitly manufactured fentanyl, increased by 38.4%. OD deaths have continued to accelerate as a result of disruptions caused by the pandemic [1,2]. According to provisional data for 2020 from the Centers for Disease Control and Prevention (CDC), US drug OD deaths increased by almost 30% to a record 93,331 deaths during the pandemic year of 2020 [3], with the highest number of deaths occurring amongst those aged 35-44 years [4]. Interventions involving OD response training, including use of the opioid antagonist naloxone, have been a critical part of the national response to the crisis. Several Food and Drug Administration (FDA)-approved products (eg, Narcan Nasal Spray) have made the administration of naloxone easier for laypeople and professional first responders by reducing the risk of needle stick injuries (NSIs) [5-11]. Recent large-scale strategies to address the opioid crisis have invoked a greater role for law enforcement, including being trained in OD response and naloxone administration. For example, researchers [12-14], health advocacy organizations, and government agencies, including the US Department of Justice [15], emphasize the need to implement programs that address the role of law enforcement in shaping health risks among people who use drugs (PWUD) [16-18].

Despite the widely perceived benefits of engaging law enforcement in OD response, considerable barriers limit the uptake of policing practices that could further public health goals [19,20]. Past research has demonstrated that police officers experience an elevated risk of NSIs emanating from routine contact with people who inject drugs (PWID), which, in turn, can lead to infectious disease acquisition. Although the risk of acquiring HIV from NSIs is low [21,22], the perceived risk is high. Concern about NSIs and broader risks related to infectious disease exposure contribute to already elevated levels of stress and burnout among the police. For instance, Strathdee et al [13] noted that of 803 officers working in Tijuana, Mexico, 666 (83%) felt that NSIs carry a level of risk analogous to a gunshot wound. Additionally, widespread sensational and misleading information in news media about incidental fentanyl exposure may reduce the likelihood an officer would intervene in an OD situation [23,24].

Good Samaritan laws providing legal immunity to OD responders who administer naloxone vary across US states. However, these laws are often not well understood, including by police officers. For example, police officers are not always aware of their protection from liability and the protection offered to bystanders [25]. Finally, persistent accounts of police unions filing “unfair labor complaints” because officers have been told to carry naloxone can be an additional barrier, even for police officers who want to carry the life-saving medication [26].

The choices that the police make also have serious implications for the relative harm and safety of the PWID whom they confront. Several studies indicate that policing strategies related to arrests for drug possession and decisions made therein about the confiscation of syringes can influence where, with whom, when, and how PWID consume drugs [27-33]. This aggravates the drug use risk environment by pushing use further to the social and geographical margins (eg, back alleys and shooting galleries). In these settings, risky consumption practices, such as syringe sharing and rushed injection, are more likely, while ODs are less likely to be witnessed [34-37]. Law enforcement may also serve as a barrier to the uptake of syringe exchange services or drug treatment, as the police have frequently been observed to obstruct the transport of syringes, harass treatment program patients, interfere in program operations, and aggressively surveil these programs, which deters people who use opioids (PWUO) from accessing them [38-44]. Racial gradients in police encounters among PWID also suggest that policing practices can be a structural driver in injection-related risk and disease acquisition [45]. Stigma against PWUO and PWID has also been common in press reports citing police chiefs and sheriffs for refusing to allow their officers to carry and administer naloxone [46,47]. A recent survey revealed that some police officers, particularly those who responded to the most OD emergencies, expressed negative attitudes about naloxone administration, drug treatment, and their role in handling drug-related OD emergencies [48].

Despite the barriers described, engaging law enforcement in opioid OD prevention and response initiatives is critical to minimizing some of the secondary harms related to commonplace policing practices [49-51]. Although some overdose education and naloxone distribution (OEND) training initiatives with law enforcement have had mixed results [48,52,53], police education programs, including our own online curricula [54,55], have shown promise in precipitating procedural and attitudinal changes related to substance use. Previous research using the Safety and Health Integration in the Enforcement of Laws on Drugs (SHIELD) model developed by another member of our team (author LB) demonstrated that police officers are especially receptive to education on working with at-risk groups when bundled with occupational safety messages that highlight their own risk of acquiring HIV, hepatitis C virus (HCV), and hepatitis B virus (HBV) from a needle stick and other harms [14,22,30,56]. Police education programs also can address pervasive misconceptions about addiction, and medication for the treatment of opioid use disorder (OUD), as well as the efficacy of harm reduction programming [57] and accurate information about the risks of fentanyl exposure [57,58]. Finally, police education can disseminate promising policing protocols to divert and deflect PWUD to lifesaving health and supportive services in lieu of arrest. Reducing the confrontational nature and frequency of contact with law enforcement is especially salient in the context of racial disparities in US drug law enforcement, although the footprint of the police’s role in treatment and other service navigation remains controversial [18,59].

Despite the promise of police education programs to reduce risky—and encourage protective—behavior, no previous study has longitudinally assessed the impact of such an intervention in the U.S. The Opioids and Police Safety Study (OPS) closes this gap with a focus on Pennsylvania (PA), Vermont (VT), and New Hampshire (NH)—3 states with some of the highest rates of OD in the U.S. (PA, 39.9/100,000; VT, 22.8/100,000; and NH, 31.1/100,000) [60,61]. Since the early years of the current opioid crisis in the U.S., PA, VT, and NH have experienced OD mortality at rates far exceeding national averages and those rates continue to rise alarmingly [62,63].

Delivering easily accessible web-based educational interventions to overcome barriers to OD response among law enforcement is particularly important in rural areas and states experiencing the highest opioid-related mortality rates. The study team has demonstrated the effectiveness of their online computer-assisted instructional course on GetNaloxoneNow.org (GNN) [54,55], which promotes learning by presenting information that requires active responding to queries or situational scenarios [64,65]. Use of feedback and remediation functions as a computer-based coach, allowing users to evaluate progress [66]. All 50 states have utilized the module, either formally when approved by state officials or informally when individuals within the state take it on their own. Accordingly, the protocol leverages existing capacity within the states while investigating potential differences between major metropolitan areas and more rural and geographically isolated areas.

Using a mixed methods design, organized around a pragmatic trial design, this study will achieve the following objectives: (1) adapt a web-based occupational risk reduction (ORR) curriculum to add to a web-based OD response and naloxone training platform, the GNN First Responder Training; (2) describe naloxone use patterns, OD response experiences, and attitudes related to illicit opioid use among a sample (N=300) of police officers in PA, VT, and NH, trained via the GNN platform; (3) evaluate the relative effectiveness of GNN + ORR compared with GNN + COVID-19 (with respect to the rates of carrying naloxone while on/off duty, rates of OD response in which naloxone is/is not administered, number of referrals to treatment, number of syringes confiscated, and rates of information sharing with OD survivors and others), as well as analyze mediators and moderators of efficacy; and (4) document the range of psychosocial mechanisms underlying participant OD response engagement postintervention.

Accordingly, our hypotheses include the following: Officers enrolled in the experimental arm will carry naloxone both while on duty and while off duty at greater rates than officers in the control arm of the study. In addition, officers in the experimental arm will also have higher rates of OD response in which naloxone is administered, a lower rate of confiscating syringes, and a higher rate of information sharing with OD survivors and others.

### Conceptual Framework

**Figure 1:**
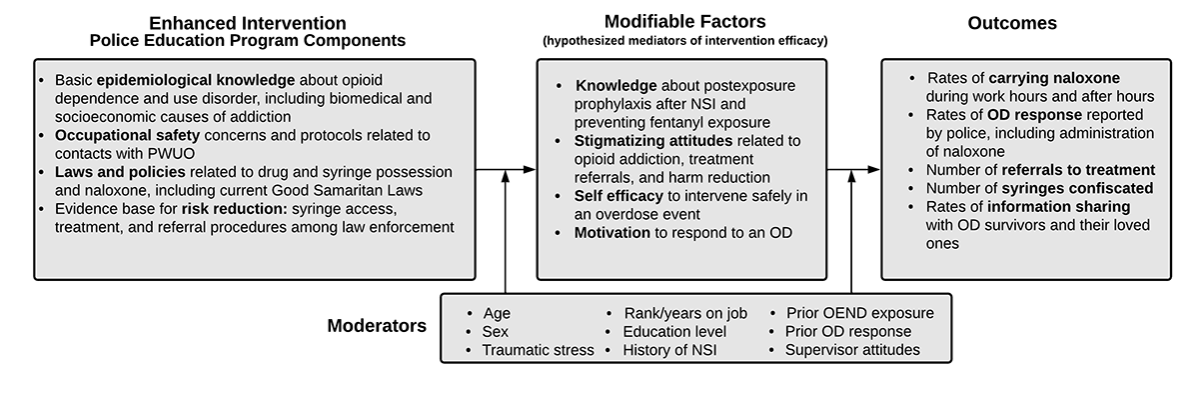
Overarching conceptual model. NSI: needle stick injury; OD: overdose; OEND: overdose education and naloxone distribution; PWUO: people who use opioids.

The study is guided by an integrated conceptual framework comprising risk environment theory (RET) and the elaboration likelihood model of persuasion (ELM). Together, these models are ideally suited to identifying and contextualizing the barriers to and facilitators of OD response (see Figure 1) and presenting strong arguments *in terms of police safety* about the value in promoting risk reduction practices and treatment among PWID [67].

*RET* defines a risk environment as “the space, whether social or physical, in which a variety of factors exogenous to the individual interact to increase vulnerability” [68]. RET specifically guides our understanding of how policing practices at the “micro” environment or community level have direct influences over the behavior, perceptions, and health outcomes of PWUO and of PWID. These practices may also increase occupational health risks for police officers. Similarly, “meso” level policy and legal statutes influence the attitudes and practices of police officers, as do “macro” level forces, such as the US war on drugs via the criminalization of drug use and the arrest and incarceration of drug users who suffer from addiction [26,69,70].

The *ELM* guides the adaptation of the ORR curricula. This model emphasizes the importance of intrinsic motivation in modifying attitudes or shaping behavior through interventions grounded in persuasion and argumentation. Guided by this theory, the ORR intervention will seek to appeal to law enforcement personnel through appeals to their own self-protection, while also highlighting the agency and utility of the police in combating the opioid crisis. These approaches take the form of strong arguments within the ELM, as they are far more likely to create favorable thoughts than what might be considered weak arguments [71,72] grounded in moral assertions about the value of PWID to society, the importance of compassionate community policing, or the disease model of addiction, for example. Within the ELM, the influence carried by an argument is also dependent upon the extent of personal involvement that the audience assumes with regard to the topic [72,73]. The proposed ORR intervention will therefore feature the voices and experiences of active police personnel and will highlight the ways in which policing practices more aligned with public health agendas are fundamentally safer for the police and the communities they serve.

## Methods

### Study Design

The protocol proposed here is a pragmatic trial design [74,75]. Pragmatic trials, unlike more conventional randomized controlled trials, are ideally suited to scenarios in which it is inefficient or unethical to isolate intervention components and compare their efficacy in an explanatory trial involving a placebo control [76].

### Intervention

The GNN First Responder Training (both experimental and control; 45 minutes) (1) explains why first responders need education and tools to reverse opioid ODs, (2) teaches and demonstrates (using animated scenarios, graphic sequences, and narration provided by professional voice actors) how to effectively respond to an opioid OD in accordance with the American Heart Association, and (3) describes barriers to calling 911 and the purpose and content of Good Samaritan laws. A detailed question-and-answer session and a posttest reinforce learning.

The OPS (experimental only; 50 minutes) provides web-based ORR training for police in 49 slides, including 8 filmed videos (police officers, physicians, syringe service program staff, and a person in recovery). The training is delivered online, with secure access only for enrolled study participants.

- Module 1 (NSIs): Teaches occupational NSI risk reduction and prevention of bloodborne disease, such as HIV, HBV, and HCV, and provides a protocol for what to do if stuck with a needle. Proper search techniques, confiscation, and the importance of clear communications are emphasized by a police officer, who also shares their own experience with an NSI and demonstrates the proper technique for preventing NSIs during a search in videos created for the training.
- Module 2 (Overdose scene safety): Addresses fentanyl potency, common myths about the risk of fentanyl exposure, how to protect oneself from accidental exposure, and what to do if an officer is exposed. A video developed for the US Customs and Border Protections is utilized in this section and clearly addresses the elevated concern law enforcement officers feel about fentanyl exposure, while simultaneously addressing the best practices in the case of suspected fentanyl exposure.
- Module 3 (Workplace wellness, stress, burnout, and compassion fatigue): Describes the mental and emotional impact of being on the front lines of the opioid crisis and how to recognize trauma and secondary trauma and provides resources for those who may be struggling. This module also presents alternatives to arrest strategies, the importance of evidence-based medication to treat OUD, and the utility of harm reduction programs in reducing recidivism and police burden.

The *COVID-19 and Police Safety Training* (control only) takes approximately 25 minutes to complete and includes 22 slides, also narrated by a professional voice narrator.

### Recruitment and Enrollment

#### Total Number of Study Participants

- Pilot: In total, 10 officers will participate in 1 of 3 hour-long online (Zoom) focus groups after viewing presentations of the content and length of the intervention and survey measures (45-minute presentations) and will provide feedback on the measures.
- Full study: In total, 300 police officers from PA, VT, and NH will be enrolled and subsequently randomized (n=150 officers in each study arm) to take either the COVID-19 control or the OPS experimental training. Both groups will then be invited to fill out quarterly online surveys over 12 months. In addition, 40 (13.3%) of the 300 enrolled police officers will also be invited to participate in 1-hour qualitative interviews during the year-long follow-up period. Prior to the administration of these longer interviews, a subset of 20 (6.7%) enrolled police officers will take a short (15-minute) qualitative interview to help us better address recruitment challenges. A subset of 20 officers will also receive incentives for successful referrals of their peers (ie, referrals that result in study enrollment).

#### Recruitment Protocol

Recruitment will focus on precincts in select PA, VT, and NH counties with high OD mortality rates. Those precincts agreeing to participate will be sent a package containing the recruiting flyers that will note the principal foci of the intervention as well as the incentives and time frame. The flyer displays the URL for the OPS secure data collection portal hosting the assessment instruments. Each participating station will also be provided with refillable cash cards supplied by the controlled trial (CT) Payer. A distinct identification number displayed on each CT card will be used by each study participant to enroll in the study and enable them to receive incentives. Although recruitment efforts initially focused on PA counties only, and included some in-person recruitment efforts, due to the COVID-19 pandemic, recruitment shifted to remote only and expanded to VT and NH as well.

#### Study Randomization

It should be noted that to avoid contamination resulting from having participants within the same precinct participating in different conditions, all members of the same participating precinct will be assigned to the same arm. Therefore, half of the precincts will be randomly assigned to the control arm and the other half to the experimental arm. Assignments are made at the initiation of online enrollment.

#### Follow-up

Participants will be asked to enter responses for the quarterly web-based survey at their earliest convenience after receiving an email reminder. Once they enter their unique identification code on a secure website that hosts the quarterly survey, participants will read questions that prompt them to enter multiple-choice responses from their keyboard or phone keypad. All questionnaires will be computerized such that participants in all study conditions can complete them using any mobile device (phone or computer) with an internet connection. Participants will complete the quarterly follow-up survey online using a unique login ID and password. Delivering surveys via a computer ensures that questionnaire administration is more consistent and accurate, data processing is eliminated, and the lag time between the data collection and data analyses phases is reduced.

### Measures

#### Baseline Data

The survey instrument is outlined in Table 1. This instrument will also assess critical demographic and psychosocial factors that could potentially moderate the intervention efficacy (ie, prior experiences with opioid-related OD, experiences with naloxone, and length of time serving as police officers). Once participants log in to the portal for the first time, complete the informed consent sections, complete their baseline survey, and complete the assigned training modules, their cash cards will be automatically credited with US $75.

**Table 1:** Measures for baseline and follow-up online survey instruments.

Measure			Description
**Outcomes**			
	Behavioral outcomes in policing procedure	Days in the past 30 days during which participants had naloxone available and carried during work Days in the past 30 days during which participants had naloxone available and carried outside of work hours Days in the past 30 days during which participants responded to an OD^a^ event, attempted to intervene, or administered naloxone Referrals to evidence-based or other drug treatment or social services made during the past 30 days Number of episodes involving syringe confiscation in the past 30 days with/without a proper technique Number of episodes involving drug confiscation with/without a proper technique	
**Hypothesized mediators of intervention efficacy**			
	Knowledge related to NSIs^b^ and treatment	Participant familiarity with the proper technique for dealing with contaminated injection equipment Participant awareness of postexposure prophylaxis (PEP) and its uses Participant awareness of the risk of fentanyl exposure and the proper technique for dealing with synthetic opioids	
	Knowledge about illicit fentanyl and analogues	Participant familiarity with fentanyl-class substances, including more recent analogues and their potency Participant familiarity with the best practices related to policing PWUO^c^ and PWID^d^ who may be carrying heroin contaminated with fentanyl-class substances (best practices derived from the Centers for Disease Control and Prevention [CDC] and Drug Enforcement Agency [DEA] curricula)	
	Opioid-related OD knowledge	Questions related to the ability to recognize and respond to an opioid-related OD, with/without naloxone adapted from the Opioid Overdose Knowledge Scale (OOKS) [77]	
	Willingness to intervene in opioid OD	Questions related to willingness, confidence, and preparedness to intervene in an opioid-related OD, adapted from the Opioid Overdose Attitudes Scale (OOAS) [77]	
	Stigma	Perceived mental health–related stigma, adapted from addiction/OUD^e^ [78] and the OOAS [77]	
	Motivation	Autonomous motivation to intervene in an opioid OD; motivation to refer PWID/PWUO to treatment—both adapted from the Situational Motivation Scale [79]	
**Potential moderators of intervention efficacy**			
	Background variables	Age and sex, adapted from the National Survey on Drug Use and Health (NSDUH) [80] Law enforcement rank and years on the job Perceived attitudes/expectations of peers, the chief, and other supervisors	
	Traumatic stress	Traumatic stress symptomatology short form checklist (Posttraumatic Stress Disorder Checklist for the *Diagnostic and Statistical Manual of Mental Disorders, Fifth Edition* [PCL-5]) [81,82]	
	Prior OD and naloxone exposure history	Prior instances of witnessing an opioid-related OD Prior instances of responding to an opioid-related OD Prior uses of naloxone to reverse an opioid-related OD	
	Past NSIs and synthetic opioid exposure	Prior incidence of an NSI or feared contact with contaminated sharp or other injection equipment Prior incidence of fentanyl exposure or feared contamination with synthetic opioids	

^a^OD: overdose.

^b^NSI: needle stick injury.

^c^PWUO: people who use opioids.

^d^PWID: people who inject drugs.

^e^OUD: opioid use disorder

#### Quarterly Follow-up Surveys

The study’s web portal will automatically generate an email notification 90 days after the completion of the first, baseline survey, informing the participant that they are eligible to access their next short online survey and receive the US $40 transfer to their cash card. Upon completion of each survey, each participant’s next notifications will be automatically scheduled to be sent 90 days later.

#### Short Qualitative Interviews

Short (15-minute) qualitative interviews will be administered via phone or Zoom to a subsample of 20 (6.7%) of 300 study participants during times that are convenient to the participants, once. Interviews will be recorded using a handheld digital recording device, and US $25 will be transferred to their cash card upon completion of the interview.

#### In-depth Qualitative Interviews

In-depth interviews will be administered via phone or Zoom to a subsample of 30 (10%) of 300 study participants during times that are convenient to the participants, once or twice during the 1-year follow-up period. Interviews will be recorded using a handheld digital recording device and will last roughly 1 hour, and US $50 will be transferred to their cash card upon completion of the interview.

### Ethics Approval

The study was approved by the New York University Institutional Review Board (IRB-FY2019-3315). The study was registered on ClinicalTrials.gov (NCT05008523) and approved on June 21, 2021. Enrollment began on January 22, 2021, prior to the approval date due to technical difficulties we experienced with the clinical trial registration website. Delays due to the COVID-19 pandemic and the national spotlight on policing practices in the U.S. posed numerous challenges to recruitment, including minimal enrollment prior to the approval of our clinical trial on ClinicalTrial.gov. To address these challenges, we added 2 sites (VT and NH) after commencing enrollment. Additional changes to our methodology—all of which were approved by the New York University Institutional Review Board (Washington Square Campus)—included the addition of a short qualitative interview (15 minutes) with a subset of approximately 20 (6.7%) of the 300 study participants to better understand and address recruitment challenges and the addition of referral incentives for study participants. There were no changes to our attitudinal and behavioral outcome measures once enrollment commenced in January 2021.

### Analysis

#### Baseline Data

Descriptive analyses will describe the sample on each of the aforementioned constructs to provide an understanding of the experiences, knowledge, and perceptions of this sample of law enforcement personnel. A series of bivariate analyses will also be used to examine how different demographic and psychosocial factors correlate with attitudes toward substance use and different degrees of OD knowledge. These analyses will build upon the team’s previous research, allowing us to carefully examine how preparedness to intervene in an OD emergency might relate to different sociodemographic and experiential factors. The results of these initial analyses will help inform subsequent analyses. Following the randomization of the sample into 1 of the 2 study arms, differences on each key construct at baseline and between the 2 study conditions/groups will be assessed to determine whether there are any statistically significant differences that must be accounted for in subsequent analyses. This will also help the study team identify which constructs produce greater variance in this sample.

#### Analysis of Intervention Efficacy Over Time

**Figure 2:**
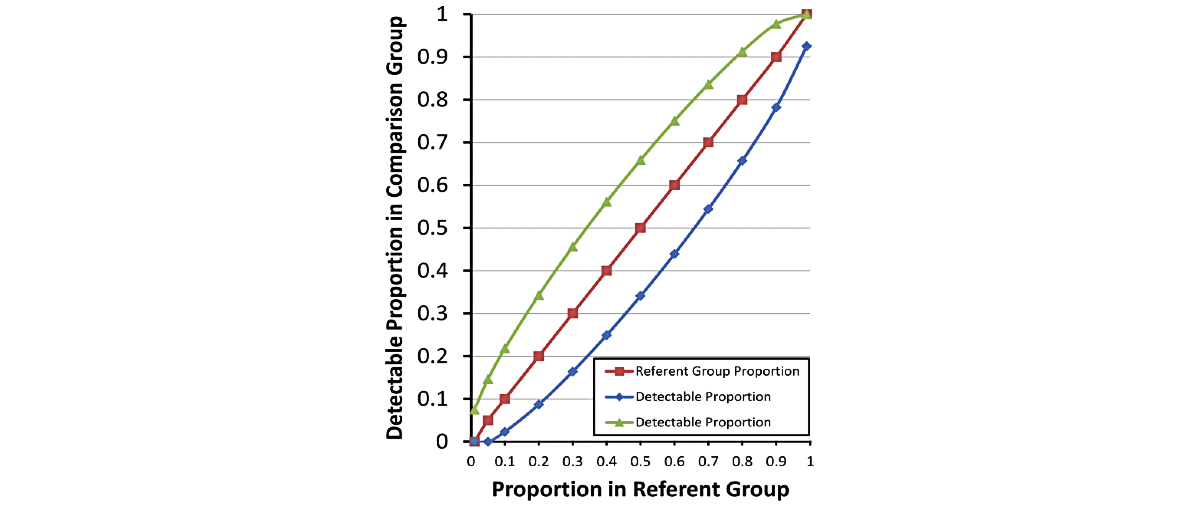
Statistically significant differences in proportions of participants achieving study endpoints between intervention and control arms.

Generalized linear mixed models will be used to analyze behavioral outcomes indicated in Table 1 and Figure 2. Overall tests of the time effect, group effect, and group-by-time interaction will be followed by contrasts of the groups at a particular point in the follow-up period (analogous to independent-sample *t* tests) and contrasts of 2 of the 5 assessment points within 1 group (analogous to paired *t* tests) to determine when the groups were different and when significant changes occurred within each group. Data from the monthly follow-up assessments will be used to assess the extent to which initial behavior changes are sustained over time. In particular, differences in behavioral outcomes between each subsequent monthly time point and baseline will be highlighted. Depending on distributions of risk behaviors, we will consider binary, Poisson, zero-inflated Poisson, negative binomial, and 2-part models.

#### Confounding Covariates

Randomization will create groups that are balanced in all characteristics, but meaningful imbalance among arms may occur by chance. Consequently, we will use multivariate analyses for each study endpoint to assess and control for confounding covariates. Baseline demographic factors and psychosocial parameters found or hypothesized in previous research to be associated with each study endpoint will be examined as possible confounders by entering each one individually, together with group assignment (study arm), into a logistic regression model. Factors changing the effect estimate for group assignment by 10% or more will be considered confounders and retained in a final multivariate model.

#### Mediation and Moderation Analyses

**Figure 3:**
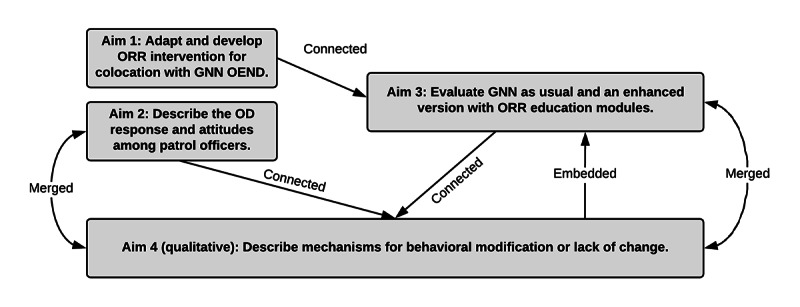
Interrelationship of study aims. GNN: GetNaloxoneNow.org; OD: overdose; OEND: overdose education and naloxone distribution; ORR: occupational risk reduction.

One of our primary analysis goals is to determine whether there are statistically significant differences in our key study outcomes (eg, are there significant differences in behavioral outcomes in policing procedures?) between our control and experimental groups. Our proposed set of analyses here will seek to evaluate these differences. All psychosocial parameters hypothesized to have a potentially mediating or moderating impact on intervention effectiveness (see Figure 3 and Table 2) will be measured at baseline and monthly follow-ups to determine whether changes in these parameters during the intervention affect the likelihood of achieving study endpoints. To evaluate effects of the intervention on key study outcome variables, while considering the potential mediating effects of psychosocial variables, we will conduct a series of path analyses using Mplus modeling software (Muthén & Muthén) [83], which accommodates categorical and continuous observed and latent variables [84] Using robust mean- and variance-adjusted weighted least squares (WLSMV) estimation, models will be fit that include paths from the intervention condition to potential mediators as well as paths from the mediators to the study endpoint. Mediated (ie, indirect) effects will be estimated by multiplying the coefficients for component direct effects (eg, the direct effect of the intervention on treatment self-efficacy × the direct effect of treatment self-efficacy on initiating treatment). Bootstrapping methods will be used to derive interval estimates of the mediated effects. Bootstrapping approaches to indirect effect inferences [85] take a random sample from the data with replacement numerous times (eg, 50,000 times) and use the variability in the statistic from sample to sample to construct an interval estimate [86]. Such estimates will provide useful insight into which aspects of our intervention are responsible for its effects, by allowing us to assess whether and how changes in mediators accounted for observed differences in outcomes.

Table 2 summarizes the main contributions of the proposed study, both to practical efforts to remediate the opioid crisis and to a broader scientific understanding of naloxone programs and educational interventions targeting law enforcement personnel.

**Table 2:** Study implications and impacts.

Gaps in extant research	Policy/intervention implications of findings
Studies on law enforcement interventions with naloxone in OD^a^ events are sparse and limited to pre- and posttests of knowledge and attitudes.	Study findings from baseline data will provide important indicators of police engagement with the topic of opioid-related OD in terms of policing behaviors and personal attitudes.
Studies on police officers’ willingness to intervene in an opioid-related OD, in relation to their attitudes toward opioid misusers, are also sparse and understudied.	Baseline findings will provide a knowledge base regarding key obstacles to and facilitators of the willingness and preparedness of the police to administer naloxone and related risk reduction practices.
The relationship of first-hand OD experiences with attitudes toward PWUO^b^/PWID^c^ is unstudied.	Baseline findings will establish potential correlations between background experiences with OD and current attitudes.
Current easily accessed OEND^d^ interventions for law enforcement do not include modules related to ORR^e^ during engagement with PWUO.	This study will be the first to prospectively examine the impact of ORR training to align law enforcement with public health goals in relation to the opioid OD crisis.
Training of law enforcement personnel does not commonly engage issues of stigma related to PWUO/PWID.	The study will assess the potential for behavioral and attitudinal modification to result from an emphasis on police safety and strong argumentation about how treatment (and referrals to treatment) and risk reduction practices protect the police.
Training tends to be limited to knowledge and confidence to intervene and is not aimed at increasing the willingness to intervene or greater engagement.	Study findings will provide empirical evidence to warrant scale-up of police education programs to cover ORR and best-practice engagement with PWUO and PWID.
ORR training protocols for law enforcement are not colocated with OEND.	Findings and enhanced ORR/OEND training curricula will be disseminated directly to law enforcement and public health professionals allowing for rapid implementation of new training protocols and curricula.
Psychosocial mechanisms underlying changes in OD knowledge and the willingness to intervene, as well as changes in stigmatizing attitudes toward opioid misusers, are not well understood.	Longitudinal (12-month) follow-ups posttraining will provide an evidence base for changes in knowledge, the willingness to intervene, and stigmatizing attitudes toward opioid misusers.
Police attitudes and practices related to PWUO/PWID are likely grounded in personal experience, such that the same event (eg, witnessing or intervening in an opioid OD emergency with or without naloxone) may compel changes in attitudes and behavior in some officers and not in others.	Qualitative research will aid in the interpretation of study findings, leading to greater specificity in terms of the psychosocial processes involved and, therefore, greater utility to law enforcement officers who frequently come in contact with opioid-dependent populations during and after OD events.
The experiences and perceptions of law enforcement personnel are not well represented in the scientific literature on OD in the current opioid crisis.	Dissemination of experiences and perceptions of law enforcement personnel will support policy and protocol changes in respect to OD-related experiences among law enforcement.

^a^OD: overdose.

^b^PWUO: people who use opioids.

^c^PWID: people who inject drugs.

^d^OEND: overdose education and naloxone distribution.

^e^ORR: occupational risk reduction.

#### Sample Size and Power Considerations

Using standard methods [87] for computing power calculations, with α=.05, and with the reasonable goal of detecting a medium effect size (*d*=0.50), we utilized G*Power (version 3.1) and determined that a randomized trial comparing 2 study arms with 150 (50%) participants in each arm will have more than 80% power that will subsequently allow us to detect statistically significant differences outcomes between our 2 groups. This proposed sample size will allow us to reasonably achieve our study aims.

#### Missing Data

In line with other research collecting online survey data, we anticipate that there may be some missing data among our survey data, but that falls within reasonable expectations for studies with this kind of study design. To address the possibility of some missing data in our analyses, we will use group mean imputation, a common estimation method utilized in survey data [88].

#### Analysis of Qualitative Interview Data

Transcribed audio-recorded interviews (N=60) will be analyzed following Lewis et al’s [89] 5-step framework analysis approach [90]. A priori qualitative constructs include the first experience with OD, personal experience with OD, naloxone knowledge, naloxone training, experience with OD and naloxone, naloxone costs and benefits, dual-role challenges, other kinds of challenges, attitudes toward PWUD and stigma, concerns about health risks, personal values and institutional environment, the opioid crisis, and COVID-19.

### Mixed Methods Synthesis

As depicted in Figure 3, the study will join qualitative and quantitative data sources at a number of key intersections throughout. Mixed methods studies in public health conventionally focus on multiple (and potentially interacting) determinants of health-related outcomes by locating points of articulation between different theoretical and methodological approaches [91-98]. Our mixed methods strategy has multiple “points of interface” [99] designed to yield a dialogue between data sources [100]. The National Institutes of Health (NIH) Best Practices for Mixed Methods Research [101] distinguishes 3 primary mechanisms for integration: merging, connecting, and embedding.

## Results

### Expected Outcomes

The OPS will be the first study to longitudinally assess the impact of a web-based opioid-related ORR intervention for law enforcement in the U.S. Findings from our randomized pragmatic clinical trial will provide evidence as to whether our experimental training actually removes barriers to life-saving police engagement with PWUO/PWID by focusing both on the safety of law enforcement and on evidence-based and best practices for working with persons at risk of an opioid OD. In sum, we expect our simultaneous embedded mixed methods approach will provide empirical evaluation of the diffusion of a naloxone-based response among law enforcement.

### Expected Timeline

Despite the fact that COVID-19-related precautions and the national spotlight on policing practices hindered study participation, we expected our baseline data collection to be complete (with a sample of 300 police officers) by the end of 2021, with a 1-year follow-up for each study participant, ending in December 2022.

## Discussion

### Summary

OD deaths are currently the largest cause of accidental deaths in the U.S., and opioid-related OD deaths constitute the overwhelming majority of these deaths. To address the epidemic, a knowledge base for effective law enforcement interventions is both necessary and urgent. This proposed study is designed to provide a knowledge base regarding key obstacles to and facilitators of the willingness and preparedness of the police to administer naloxone and the related risk reduction practices, and evaluate the efficacy of a web-based opioid-related occupational safety and risk reduction curriculum.

### Strengths and Limitations

The OPS is the first study to longitudinally assess the impact of an opioid-related ORR intervention for law enforcement in the U.S.

Our randomized pragmatic clinical trial aims to remove barriers to life-saving police engagement with PWUO/PWID by focusing both on the safety of law enforcement and evidence-based and best practices for working with persons at risk of an opioid OD.

Our simultaneous embedded mixed methods approach will provide empirical evaluation of the diffusion of the naloxone-based response among law enforcement.

However, COVID-19-related precautions and the national spotlight on policing practices may hinder study participation.

### Conclusion

Findings from this study will be applied to the development and implementation of effective interventions for police officers aimed at harmonizing law enforcement practices with public health goals. Study products (training curricula) will also be disseminated nationally.

## Abbreviations

ELM: elaboration likelihood model of persuasion
GNN: GetNaloxoneNow.org
HBV: hepatitis B virus
HCV: hepatitis C virus
NSI: needle stick injury
OD: overdose
OEND: overdose education and naloxone distribution
OPS: Opioids and Police Safety Study
ORR: occupational risk reduction
OUD: opioid use disorder
PWID: people who inject drugs
PWUD: people who use drugs
PWUO: people who use opioids
RET: risk environment theory
